# Visceral leishmaniasis misdiagnosed as an upper respiratory infection and iron-deficiency anemia in a 20-month-old male patient: a case report

**DOI:** 10.1186/s13256-024-04356-y

**Published:** 2024-01-31

**Authors:** Davit G. Chakhunashvili, Konstantine Chakhunashvili, Eka Kvirkvelia

**Affiliations:** 1 Department of Pediatrics, Alte University, Tbilisi, Georgia; 2 Department of Pediatrics, The University of Georgia, Tbilisi, Georgia; 3 Department of Gynecology, Caucasus University, Tbilisi, Georgia; 4 Children’s Clinic After I. Tsitsishvili, Tbilisi, Georgia

**Keywords:** Visceral Leishmaniasis, Pancytopenia, Bicytopenia

## Abstract

**Background:**

Visceral Leishmaniasis should be suspected in every patient with a history of splenomegaly, fever, and pancytopenia. It is one of the most dangerous forms of infection and prompt recognition is the key to positive outcome.

**Case presentation:**

A 20-month-old Caucasian male patient was brought to our hospital as an outpatient with the complaint of persistent fever, which did not improve with empiric antibiotic treatment (> 96 hour after the initial dose). The antibiotic treatment had been prescribed by primary care physician at polyclinic, who also referred the patient to hematologist due to anemia, who prescribed iron supplement. Despite multiple subspecialist visits, bicytopenia was, unfortunately, left unidentified. Upon physical examination no specific signs were detected, however, spleen seemed slightly enlarged. Patient was admitted to the hospital for further work-up, management and evaluation. Abdominal ultrasound, complete blood count and c-reactive protein had been ordered. Hematologist and infectionist were involved, both advised to run serology for Epstein-Barr Virus and Visceral Leishmaniasis. The latter was positive; therefore, patient was transferred to the specialized clinic for specific management.

**Conclusion:**

Both in endemic and non-endemic areas the awareness about VL should be increased among the medical professionals. We also recommend that our colleagues take the same approach when dealing with bicytopenia and fever, just as with pancytopenia and fever. The medical community should make sure that none of the cases of fever and pancytopenia are overlooked, especially if we have hepatomegaly and/or splenomegaly.

## Introduction

Pancytopenia is an important clinical-hematological presentation, in which all three key peripheral blood lineage is reduced, including red blood cells, white blood cells, and platelets [1]. Prompt recognition of the underlying cause of pancytopenia can significantly affect the morbidity and mortality of children, although bicytopenia might be just as dangerous and might devolve into pancytopenia, it is often overlooked and not as many papers are published about it [1, 2]. Bone marrow aspiration and biopsy are highly recommended in patients with pancytopenia especially if the underlying cause is unknown [3]. Visceral Leishmaniasis (VL) should be suspected in every child with a history of splenomegaly, fever, and pancytopenia, this course of events is mainly determined by parasitic invasion of macrophages and their transmission to liver, spleen, bone marrow, which will lead to bicytopenia and ultimately to pancytopenia [4, 5]. 50,000 to 90,000 new cases are expected to occur each year [6]. Risk factors such as urbanization, humidity, altitude, rainy seasons, and socioeconomic factors should be kept in mind [7]. The case we are reporting involves a patient with fever and bicytopenia, the latter was overlooked in this encounter, and it devolved into pancytopenia, which is much more published and talked about in the research/medical community.

## Case presentation

### Patient information

A 20-months-old Caucasian male patient was brought to the hospital as an outpatient. The main complaint was persistent fever and no improvement despite the antibiotic treatment. Parents mentioned that they visited their primary care physician at the polyclinic, who had prescribed antibiotic therapy, and were forwarded to the hematologist, who prescribed ferrous sulfate. The patient had baseline complete blood count (CBC) analysis on their hand (Table 1). Parents mentioned no relevant travel history, the solid feeding has been successfully initiated at 6 months of age and by the time of this event the daily ration included well-balanced diet.

**Table 1:** This table includes results of Complete Blood Count and C-reactive protein up until the day of the discharge of the patient

	Baseline	Follow-up	In-patient	
	Day 1	Day 6	Day 8	Day 14
White Blood Cell Count (X 10^9^/L)	4	4	6.86	7.62
Neutrophil Count (X 10^9^/L)	1.48	1.04	1.5	2.36
Lymphocyte Count (X 10^9^/L)	2.12	2.48	4.7	4.57
Red Blood Cell Count (X 10^12^/L)	3.6	2.99	3.00	3.54
Hemoglobin (g/dL)	8.6	7.2	6.8	9.3
Hematocrit (%)	28.3	21.9	–	–
Mean Corpuscular Volume (fL)	79	73.2	–	–
Platelet Count (X 10^9^/L)	155	190	63	106.1
C-reactive Protein (mg/dL)	–	126.7	–	152.5

### Timeline and diagnostic assessment

On the day one after initial assessment and two days after the onset of the symptoms, the patient was diagnosed with an upper respiratory tract infection and was prescribed empiric antibiotic therapy, due to fever and high erythrocyte sedimentation rate (Table 1). The patient was also referred to a hematologist for assessment of low hemoglobin levels and was prescribed ferrous sulfate for presumed iron-deficiency anemia (IDA) by the hematologist as well as setting the follow-up visit after 2–3 weeks (Table 1).

The patient was brought to us on the sixth day from the onset of fever and more than 96 hour (4 days) after the first dose of the Amoxicillin Clavulanate. A thorough physical examination yielded the following: Heart rate (HR)—138, respiratory rate (RR)—34, oximetry—98%, blood pressure—90/55 mm of mercury (mm Hg); Skin—pallor, capillary refill—normal; Oral—no hyperemia or lesion were detected; Eyes—normal; Ear—no abnormalities were detected, tympanic membrane—normal; Chest—no retractions, lung sounds were normal and symmetrical, heart sounds were normal; Abdomen—no pain was detected, spleen seemed slightly enlarged; Joint pain—negative; Central or Peripheral Nervous System symptoms and signs—negative, consciousness was not altered; Urination—normal, no dysuria; Defecation—normal, no constipation or diarrhea. Despite the antibiotic therapy, no improvement was observed.

Taking all the facts into consideration, the patient was admitted for further evaluation and management of a possible life-threatening condition.

Initial work-up at our clinic included CBC, abdominal ultrasound, and C-reactive protein, as well as involvement of a hematologist and an infectionist. CBC was initiated to check if the levels of certain blood cell lines were deteriorating, abdominal ultrasound was initiated to check for the enlargement of spleen and liver. Due to fever, splenomegaly and bicytopenia it was concluded that we would test for Epstein-Barr Virus (EBV) and Visceral Leishmaniasis (VL) in the first place.

As soon as we received positive results for VL, the patient was transferred to the specialized hospital (Research Institute of Medical Parasitology and Tropical Medicine).

### Complete blood count

The first CBC showed decreased white blood cell (WBC) count (4 × 10^9^/L), and red blood cell (RBC) count (3.6 × 10^12^/L); Hemoglobin level was also decreased (8.6 g/dL), however, mean corpuscular volume (MCV) was normal. Hematocrit (HCT) also decreased (28.2%) (Table 1).

The second CBC showed the same WBC count (4 × 10^9^/L), RBC decreased even further (2.99 × 10^12^/L) as did hemoglobin (7.2 g/dL) and HCT (21.9%), and MCV stayed normal (Table 1).

Erythrocyte sedimentation was increased on both the first and second measurements, 44 mm/hour and 35 mm/hour, respectively.

Although, platelet count was low in both the first and second CBC results according to laboratory reference, we did not count it as thrombocytopenia since the values were not below 150 × 10^9^/L. Hence, by this time we had bicytopenia rather than a pancytopenia, but the approach remained the same.

The results continued to deteriorate on day 8 as well (Table 1). Namely, hemoglobin and platelet count continued to drop (6.8 mg/dL and 63 × 10^9^/L). Once the patient was transferred to a specialized hospital and specific treatment had been initiated.

### C-reactive protein

C-reactive protein was significantly increased on the sixth day (126.7 mg/dL) (Table 1).

### Ultrasound

On the first abdominal ultrasound, the long axis of the spleen was markedly increased (110 mm), liver sizes were normal, and no other abnormalities were found. The long axis continued to increase and was the largest on Day 7–128 mm. On the day of the discharge, the long axis was reduced to 120 mm.

### Serology

Results were negative for acute EBV infection; However, they were positive for Visceral Leishmaniasis.

### Therapeutic intervention

Before the diagnosis was confirmed, supportive treatment and empiric intravenous antibiotic therapy was initiated as soon as the patient got admitted to the hospital. After the confirmation of VL, the patient was transferred to a specialized hospital and meglumine antimoniate was initiated, which is considered first line treatment according to Georgian protocol proposed by Ministry of Health, the following tests are to be done to monitor for the side effects of the medication: CBC, AST/ALT, Amylase, Lipase and ECG. No issue was detected, supportive treatment was ongoing, however, a single RBC transfusion was required to correct the anemia.

### Follow-up

Patient is under ambulatory surveillance by the doctors at the Research Institute of Medical Parasitology and Tropical Medicine and is being followed-up every month.

## Discussion

The case is a good reminder that in setting of fever bicytopenia needs to be paid as much attention as pancytopenia usually is. There have been very few studies and reports about bicytopenia, especially, when compared to pancytopenia [2]. Therefore, this case can be an example that bicytopenia can devolve into pancytopenia and taking a closer look at the patients may assist in early detection of diseases that can present with fever, bicytopenia/pancytopenia.

VL (a severe form of Leishmaniasis), one of the world’s most dangerous infections, is caused by parasites Leishmania Donovani and Leishmania infantum, which can be transmitted via a bite from phlebotomine sand flies, the incubation period of the disease has been reported to extend from few days to 10 years [8, 9]. Once infected, protozoan parasites start to target lymph nodes, the immune system, and other reticuloendothelial organs like the liver, spleen, and bone marrow, destroying white blood cells and red blood cells [1, 10, 11]. As this disease can affect immunity as well, it is also referred to as a parasitic form of AIDS [8]. VL in children can manifest with intermittent fever and other mild symptoms or can even be asymptomatic [12]. In 25% of untreatable cases, the disease can progress into an active illness in a few months, spleen will begin to enlarge, and fever will become intermittent [12]. Prolonged fever, hepatosplenomegaly, pancytopenia, weight loss, and pallor are classic clinical features of Visceral Leishmaniasis and should be ruled out as soon as possible [12]. According to one study, 15% of children presenting with pancytopenia were infected with Visceral Leishmaniasis [8]. Amphotericin B and pentavalent antimonials are the most used drugs to treat this condition [10]. Diagnosis of VL can be made after the detection of parasites in spleen or bone marrow aspirates, or after antibody detection via serologic testing [11]. Leishmania parasite can also be detected with the help of polymerase chain reaction (PCR), which has high sensitivity and specificity [13]. For better prognosis, it is crucial to diagnose Visceral Leishmaniasis on time [4]. Malaria and acute leukemia are important differential diagnoses, as both can manifest with fever, splenomegaly, and pancytopenia [4]. Other hematological etiological factors of pancytopenia in children can be megaloblastic anemia, myelodysplastic syndrome, or aplastic anemia [14]. Cytostatic drugs and certain viral agents like Cytomegalovirus (CMV), Epstein-Barr virus (EBV), Human Immunodeficiency Virus (HIV), influenza, rubella, parainfluenza, hepatitis A, adenovirus, Parvovirus B19 etc. can also manifest with pancytopenia in children [14]. For endemic regions, tuberculosis and brucellosis can also be suspected in patients who present with fever, splenomegaly, and pancytopenia [15]. Leishmaniasis can be easily misdiagnosed as an autoimmune disease as different autoantibodies can be present in the course of the disease [15]. VL accompanied by positive antinuclear antibodies and multiorgan involvement can also be easily misdiagnosed as Systemic Lupus Erythematosus [16]. Antibodies against neutrophils and platelets in conjunction with positive direct Coombs test can be misdiagnosed as autoimmune disease [14]. Autoimmune pancytopenia like Hemophagocytic lymphohistiocytosis (HLH) syndrome should also be considered [14].

It is important to highlight that VL is one of the most dangerous and life-threatening diseases, therefore, rapid diagnosis is of the essence. Although, it is a challenge. There are a lot of delayed cases of diagnosis even in endemic areas. According to the National Centre for Disease Control and Public Health of Georgia, 35 and 20 cases of VL have been reported in 2020 and 2021, respectively. Unfortunately, it is necessary to state that one of the reasons for delayed diagnoses is neglecting the possibility of VL, such as overlooking bicytopenia, which often progresses to pancytopenia, also physical exam should be thorough not to miss enlargement of the spleen and/or liver, and it would be prudent to initiated abdominal ultrasound with follow-up CBC to monitor the disease progression especially in prolonged fever [1, 2, 14]. Literature reviews tell us that time to diagnosis after onset of the symptoms is too long, median—30 days and the peak represents 5 months [17–20].

One article reports, that a patient was diagnosed 8 days after hospital admission which represented the 28th day since the symptoms had occurred [17]. Multiple articles state that the day of VL diagnosis might range from several days (The lowest reported was 7 days) to multiple months [18, 19].

Since VL is a serious disease, it might be fatal in most patients if the correct diagnosis and treatment are not rendered soon [21]. The diagnosis can be delayed due to various objective and subjective reasons. Microscopic examination (spleen, bone marrow, lymph node) is the gold standard [22–24], but in certain cases, due to invasiveness of the procedure it might be postponed. That is why if patient has clinical presentation like VL it might be worth considering doing serological testing as soon as possible and then trusting positive results, even though it has been widely accepted that serological tests might come up positive in asymptomatic patients or in those who had the disease in the past [25–27].

One of the challenges of diagnosing VL is the vast and thorough differential diagnosis that needs to be done. Typical signs of VL include fever, hepatomegaly and/or splenomegaly, pancytopenia, however, these signs are not specific for just VL and can include myriad of problems including rheumatic, infectious, malignant disorders etc.

**Fig. 1:**
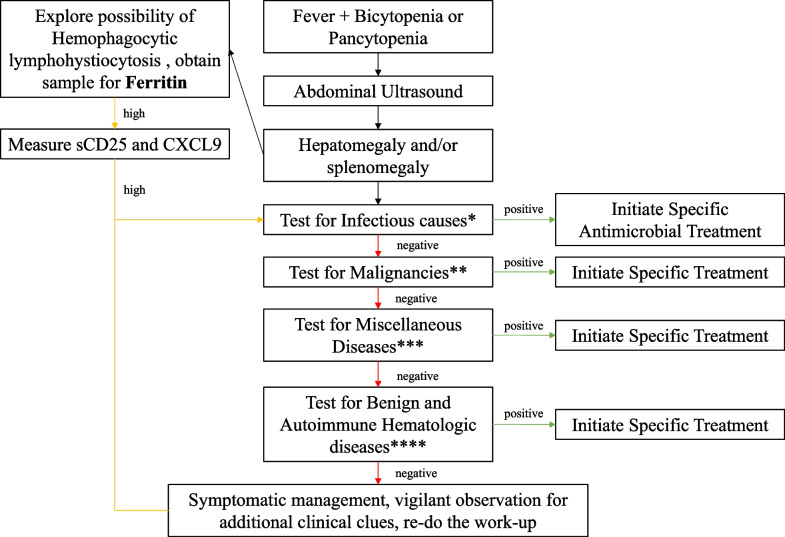
This figure depicts an algorithm that can be used in patients with fever, bycitopenia or pancytopenia, and hepatomegaly and/or splenomegaly. * Exclude Visceral Leishmaniasis, Epstein-Barr Virus, Human immunodeficiency Virus, Cytomegalovirus, Malaria, Shcistosomiasis, Histoplasmosis, Mycobacteria, Typhoid Fever, Brucellosis, Tuberculosis, Parvovirus B19, Influenza, Rubella, Parainfluenza, Hepatitis A, Adenovirus. ** Exclude Leukemia, Polycetemia Vera, Lymphoma, Myelofibrosis with Myeloid Metaplasia via bone marrow, lymph node or spleen biopsy, respectively. *** Exclude Splenic Tumors, Sarcoidosis, Gaucher’s Disease via Splenic, lymph node or bone marrow biopsy, respectively; We would also recommend testing levels of Anti-Nuclear Antibodies, not to miss out on Systemic Lupus Erythematous; Do not overlook the possibility of Systemic Juvenile Idiopathic Arthritis. **** Exclude Autoimmune Anemia and Hemoglobinopathies via direct agglutination and hemoglobin analysis/DNA tests, respectively; Explore the possibility of vitamin deficiencies

Differential diagnosis can be even harder if there are no other specific signs, symptoms, and clues (specific drug use etc.). Hence, we have come up with a comprehensive algorithm that helps not to miss important differential diagnoses (Fig. 1).

One of the things that should always be deliberated during hepatomegaly and/or splenomegaly, fever, bicytopenia/pancytopenia is HLH. We recommend always obtaining ferritin levels together with other work-up. If ferritin levels are extremely high (> 500 ng/mL), we offer to further explore the possibility of HLH (Fig. 1) [28]. Even if the diagnosis is confirmed we should still explore the triggering factor, so the investigation should continue, whilst the supportive and symptomatic treatment are initiated [29, 30]. Not obtaining Ferritin levels in the case of this patient was one of the major critical errors.

When there is no specific clue but the signs and symptoms, such as fever, bicytopenia/pancytopenia, splenomegaly one of the things that should be considered is EBV and we strongly recommend testing for the infection [32]. Obtaining samples for VL detection should not be delayed as well [33]. The possibility of HIV should also be explored, since it can be the cause for this type of clinical presentation or, if present, other non-typical causative might also be the reason for the clinical picture [34].

Rare cause—Tuberculosis—should not be overlooked in such cases, especially if it is an endemic region, just like Georgia [35]. The same can be said about brucellosis [36].

If none of the above-mentioned tests are positive, possibility of other infective agents should not be forgotten—CMV [37], malaria [38], parvovirus b19 [39], influenza [40], rubella [41], hepatitis A [42], adenovirus [43], schistosomiasis [44], histoplasmosis [45], MAV [34], typhoid fever [47]—and the samples should be tested against these microorganisms.

Next step for differential diagnosis, if none of the infective agents are confirmed, should be exclusion of malignancies, which require a more invasive approach—biopsy of bone marrow, spleen, and lymph nodes [34, 47–50]. We recommend obtaining enough specimens to test them for splenic tumors, sarcoidosis, Gaucher’s disease as well [51–53].

Although it is quite rare, especially in male patients, possibility of SLE should not be neglected and it would be prudent to check the levels of Anti-Nuclear Antibodies [54]. Another exceedingly rare disease that should also be considered is systemic juvenile idiopathic arthritis, however, since this is a diagnosis of exclusion, these assays should help us rule out other conditions [55, 56].

In case of fever, bicytopenia/pancytopenia, and splenomegaly a clinician should always keep in mind that autoimmune hemolytic anemia (AIHA) might be present; keep in mind, that various infectious agents may trigger the condition [57]. Therefore, we recommend running a direct antiglobulin test (DAT), which should be positive in case of classical presentation, but the physician should keep in mind that this does not rule out all types of AIHA [58].

There have been reports of unusual presentation of vitamin B12 deficiency presenting with fever, pancytopenia, and splenomegaly, therefore, we also recommend collecting detailed dietary history and checking the levels of vitamin B12 [59].

The case demonstrates that primary care physicians and doctors from other fields of medicine should be more vigilant, hence, they must not jump to early conclusions when a patient has a fever with pancytopenia or bicytopenia. The first issue was bicytopenia (that then devolved to pancytopenia) being unnoticed, while solely focusing on fever and attributing these events to upper respiratory bacterial infection. The second issue was for the hematologist to consider this condition as IDA, when there were objective red flags, such as fever, bicytopenia, hemoglobin levels going down and MCV staying in a normal range.

On the other hand, the case also is a good example that swift action and interventions could lead to a successful diagnosis of VL on the sixth day after the onset. Although the patient recovered, we consider that not running the ferritin test at the beginning is a major flaw on our part and we urge our colleagues not to make the same mistake.

We also recommend following the algorithm, of course, if there are no specific clinical clues (signs, symptoms, history) and we only have fever, bicytopenia or pancytopenia. However, we recommend skipping the steps if disease-specific clinical clues become apparent.

Considering the course that VL took in the patient, we strongly believe that even a 24-h delay in diagnosis might have been fatal. Therefore, this is a good case to study from and we hope it will help our colleagues worldwide not to make the same diagnostic errors.

## Conclusion

Both in endemic and non-endemic areas the awareness about VL should be increased among the medical professionals. We also recommend that our colleagues take the same approach when dealing with bicytopenia and fever, just as with pancytopenia and fever. The medical community should make sure that none of the cases of fever and pancytopenia are overlooked, especially if we have hepatomegaly and/or splenomegaly. In such cases, when there are no specific clinical clues for a specific diagnosis, we recommend pursuing the proposed algorithm.

## Data Availability

[REAGENTS/TOOLS/MATERIALS] generated in this case report are available from the corresponding author upon request.
